# Impact of Tafamidis on Delaying Clinical, Functional, and Structural Cardiac Changes in Patients with Wild-Type Transthyretin Amyloid Cardiomyopathy

**DOI:** 10.3390/jcm13133730

**Published:** 2024-06-26

**Authors:** Giuseppe Palmiero, Emanuele Monda, Federica Verrillo, Francesca Dongiglio, Chiara Cirillo, Martina Caiazza, Marta Rubino, Annapaola Cirillo, Adelaide Fusco, Gaetano Diana, Giovanni Ciccarelli, Santo Dellegrottaglie, Paolo Calabrò, Paolo Golino, Giuseppe Limongelli

**Affiliations:** 1 Department of Translational Medical Sciences, Inherited and Rare Cardiovascular Diseases, University of Campania “Luigi Vanvitelli”, 80131 Naples, Italy; g.palmiero@hotmail.it (G.P.); emanuelemonda@me.com (E.M.); fedeverrillo@gmail.com (F.V.); f.dongiglio@gmail.com (F.D.); kiaracirillo@gmail.com (C.C.); martina.caiazza@yahoo.it (M.C.); rubinomarta@libero.it (M.R.); cirilloannapaola@gmail.com (A.C.); adelaidefusco@hotmail.it (A.F.); gaetanodiana1991@gmail.com (G.D.); paolo.calabro@unicampania.it (P.C.); 2 Vanvitelli Cardiology Unit, Department of Translational Medical Sciences, Monaldi Hospital, 80131 Naples, Italy; ciccarelli.giovanni@gmail.com (G.C.); paolo.golino@unicampania.it (P.G.); 3 Sbarro Institute for Cancer Research and Molecular Medicine, Center of Biotechnology, College of Science and Technology, Temple University, Philadelphia, PA 19122, USA; 4 Advanced Cardiovascular Imaging Unit, Ospedale Medico-Chirurgico Accreditato Villa dei Fiori, 80131 Naples, Italy; santo.dellegrottaglie@mountsinai.org

**Keywords:** amyloidosis, transthyretin, tafamidis

## Abstract

**Background**: This study aimed to evaluate the effect of treatment with tafamidis on clinical, laboratory, functional, and structural cardiovascular imaging parameters at the 12-month follow-up timepoint in patients with wild-type transthyretin amyloid cardiomyopathy (ATTRwt-CM) and to assess the response to treatment in terms of disease progression. **Methods**: Patients with ATTRwt-CM undergoing treatment with tafamidis for >12 months were included. The patients underwent a comprehensive evaluation (including echocardiography, cardiac magnetic resonance imaging, six-minute walking test, assessment of quality of life, and laboratory tests) at baseline and the 12-month follow-up timepoint. Disease progression was assessed using a set of tools proposed by an international panel of experts, evaluating three main domains (clinical, biochemical, and structural). **Results**: The study cohort consisted of 25 patients (mean age of 75.9 ± 6.1 years, with 92% males). At the 12-month follow-up timepoint, an improvement in quality of life calculated with the KCCQ overall score (64 ± 20 vs. 75 ± 20, *p* = 0.002) and a reduction in pulmonary artery pressure (34 ± 10 mmHg vs. 30 ± 5 mmHg, *p*-value = 0.008) and in native T1 time were observed (1162 ± 66 ms vs. 1116 ± 52 ms, *p*-value = 0.001). Clinical, biochemical, and structural disease progression was observed in 6 (24%), 13 (52%), and 7 (28%) patients, respectively. Overall disease progression was observed in two patients (8%). **Conclusions**: This study described the impact of tafamidis treatment on clinical, laboratory, and functional parameters. Disease progression, assessed using a multiparametric tool recommended by a recent position paper of experts, was observed in a minority of patients.

## 1. Introduction

Transthyretin amyloid cardiomyopathy (ATTR-CM) is a progressive and potentially fatal disease caused by TTR misfolding and aggregation into insoluble amyloid fibrils that deposit in the myocardium, leading to infiltrative cardiomyopathy and heart failure (HF) [1,2]. Two forms of ATTR have been described: hereditary ATTR (ATTRh) caused by pathogenic variants in the *TTR* gene and wild-type ATTR (ATTRwt) where no pathogenic variant is identified [3]. The pathogenesis of ATTR amyloidosis is associated with the destabilization of the tetrameric TTR protein [4]. Tetramer dissociation is the rate-limiting step in TTR aggregation, which also requires subsequent monomer misfolding to produce soluble misfolded TTR aggregates and insoluble aggregates, including amyloid fibrils [4]. In ATTR-CM, the deposition of TTR amyloid in the myocardium leads to a progressive increase in myocardial wall thickness and impairment in diastolic function [5,6,7].

Tafamidis is a TTR kinetic stabilizer that inhibits tetramer dissociation [8]. In a randomized controlled study, namely, the Transthyretin Cardiomyopathy Clinical Trial (ATTR-ACT; ClinicalTrials.gov identifier: NCT01994889), Maurer et al. showed that treatment with tafamidis was associated with a significant reduction in mortality and cardiovascular-related hospitalization and smaller decline in the functional capacity and quality of life of patients with ATTR amyloidosis compared to placebo [8]. The post-hoc analysis of the ATTR-ACT trial and reports from single centers showed that treatment with tafamidis may attenuate the decline in cardiac function [9,10,11,12]. These studies mainly focused on basic echocardiographic parameters, with limited data regarding the impact of tafamidis treatment on improving the overall disease using a multiparametric approach, including ECG, echocardiography, cardiac magnetic resonance (CMR), biomarkers, functional capacity, and quality of life.

Recently, a consensus document from an international expert panel recommended a set of clinically feasible tools for the long-term monitoring of patients with ATTR-CM, including thresholds for defining disease progression and the frequency of measurements [13]. However, the implementation of these tools in clinical practice has never been reported.

This study aimed to evaluate the effect of treatment with tafamidis on clinical, laboratory, functional, and structural cardiovascular imaging parameters at the 12-month follow-up timepoint in patients with ATTRwt-CM and assess the response to treatment in terms of clinical, biochemical, and structural progression using the clinical tools for long-term monitoring proposed by a recent consensus document.

## 2. Methods

An observational, longitudinal, and prospective cohort design was employed. This study adhered to the principles of the Helsinki Declaration and received approval from the ethics committee of our institution. All patients provided written informed consent.

### 2.1. Study Population

The study population consisted of consecutive patients with ATTRwt-CM, who were prospectively evaluated at the Inherited and Rare Cardiovascular Disease Clinic of the University of Campania “Luigi Vanvitelli”—Monaldi Hospital (Naples, Italy) between March 2018 and December 2023. ATTRwt-CM was defined by positive 99mTc-hydroxy methylene-diphosphonate scintigraphy (i.e., grade 2 or 3 cardiac uptake) in the absence of monoclonal gammopathy or by a cardiac biopsy containing TTR amyloid in the presence of monoclonal gammopathy [14]. Genetic testing was performed to exclude a pathogenic or likely pathogenic variant in the *TTR* gene.

### 2.2. Eligibility Criteria

To be eligible for enrollment, patients had to meet the following inclusion criteria: diagnosis of ATTRwt-CM; therapy with tafamidis 61 mg once daily for 12 months; and complete clinical, biochemical, functional, and imaging assessment at baseline and follow-up evaluations. Patients diagnosed with ATTRh-CM and those without complete serial evaluations during follow-up were excluded.

### 2.3. Clinical Investigation and Data Collection

Patients who initiated treatment with tafamidis underwent a comprehensive cardiovascular assessment, including clinical history, physical examination, ECG, echocardiography, CMR, six-minute walking test (6-MWT), assessment of quality of life using the Kansas City Cardiomyopathy Questionnaire (KCCQ), and laboratory assessment. This assessment was performed at baseline evaluation (corresponding to the introduction of tafamidis treatment) and at the one-year follow-up timepoint.

### 2.4. Echocardiography

All patients underwent standard transthoracic echocardiography using a Vivid E9 ultrasound system (GE Healthcare, Horten, Norway) equipped with an M5S 3.5-MHz transducer. Two-dimensional, color-Doppler, pulsed-wave, and continuous-wave Doppler data were acquired and stored on a dedicated workstation for offline analysis (EchoPAC Version 204, GE Vingmed Ultrasound, Norway). Chamber quantification followed current recommendations [15]. The myocardial contraction fraction (MCF) was calculated as the ratio of stroke volume (SV) to myocardial volume, obtained through three-dimensional (3D) analysis and LV mass calculations [16]. LV peak systolic longitudinal strain (LS) and 2D speckle tracking (2D-ST) strain measurements were performed, and the LV global longitudinal strain (GLS) was calculated [17]. The relative regional strain ratio (RRSR) was calculated [18], and myocardial work (MW) and related indices were estimated using vendor-specific software [19]. Diastolic parameters were collected by wave and tissue Doppler, and pulmonary artery systolic pressure (PASP) was estimated [20]. The tricuspid annular plane systolic excursion (TAPSE) was measured, and right ventricular pulmonary artery uncoupling was defined using the TAPSE/PASP ratio [21]. The pulmonary artery flow and RV fractional area change (FAC) were measured, and RV myocardial deformation parameters were calculated using the Q-Analysis software package [22]. RV global and free wall longitudinal strain values were obtained. To avoid interobserver variability, echocardiographic evaluations were performed by the same operator (G.P.).

### 2.5. Cardiac Magnetic Resonance

CMR examinations were conducted on a 1.5 Tesla scanner (MAGNETOM Avanto, Siemens Healthcare GmbH, Erlangen, Germany) following standard protocols, which included late gadolinium enhancement (LGE) imaging (0.1 mmol/kg gadobutrol; Gadovist, Bayer Vital GmbH, Leverkusen, Germany) and T1 mapping using the modified Look-Locker inversion (MOLLI) sequence. CMR imaging included cine-imaging, pre- and post-contrast T1 mapping, and additional calculations of extracellular volume fraction (ECV) values [23]. CMR evaluation was performed at the time of therapy initiation ± 3 months.

### 2.6. Laboratory Assessment

Serum levels of sodium, creatinine, hemoglobin, albumin, high-sensitivity cardiac troponin I (HS-TnI), and N-terminal pro-B-type natriuretic peptide (NT-proBNP) were measured from peripheral venous blood samples in all patients using standard commercially available assays. The estimated glomerular filtration rate was calculated using the CKD-EPI equation [24]. Patients with ATTRwt-CM were staged according to the staging systems proposed by Gillmore et al. [25].

### 2.7. Quality of Life and Functional Capacity

The HF-related health status was quantified by the KCCQ score, which includes symptom frequency, physical limitations, social limitations, and quality-of-life domains [26]. The scores ranged from 0 to 100, with 100 signifying better health status. The patients completed the KCCQ guided by nurses, who were unaware of the baseline assessment.

Functional capacity was assessed using the 6-MWT. It was performed indoors using a 30-meter corridor, which was marked every 3 meters, and the turnaround points were marked with a cone.

### 2.8. Assessment of Disease Progression

Disease progression was evaluated after 12 months using a set of clinically feasible tools for the long-term monitoring of patients with ATTR-CM proposed by an international panel of experts [13]. The assessment of disease progression was performed using a set of 11 features across three main domains: clinical domain (including the assessment of clinical and medical history, NYHA class, quality of life, and functional capacity); biochemical domain (including the assessment of biomarkers and laboratory markers); and structural domain (including the assessment of ECG and echocardiographic parameters). Disease progression was defined as the presence of at least one marker in each of the three domains.

### 2.9. Statistical Analysis

Body surface area (BSA) was calculated from height and weight. The normally distributed continuous variables are described as mean ± standard deviation with two group comparisons conducted using the Student’s *t*-test. The changes in continuous laboratory or imaging parameters were assessed using the paired Student’s *t*-test. Skewed data are described as the median (interquartile range [IQR]), with two group comparisons performed using the Wilcoxon rank-sum test. The categorical variables are listed as a number (percentage), with group comparisons conducted using the χ^2^ test or Fisher’s exact test. A significance level (*p*-value) of 0.05 (two-sided test) was used for all the comparisons. All statistical analyses were performed using IBM SPSS Statistics for Macintosh, Version 27.0.

## 3. Results

### 3.1. Study Population, Functional Status, and Biochemical Parameters

**Figure 1:**
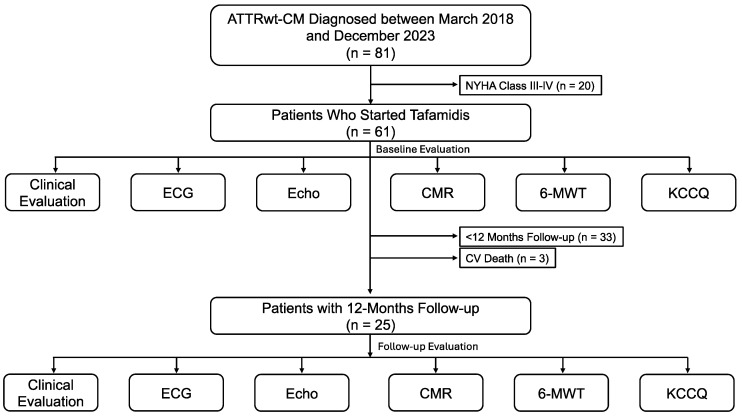
Study flow chart. Abbreviations: 6-MWT, 6 min walking test; ATTRwt-CM, wild-type transthyretin amyloid cardiomyopathy; CMR, cardiac magnetic resonance; ECG, electrocardiography; Echo, echocardiography; KCCQ, Kansas City Cardiomyopathy Questionnaire; and NYHA, New York Heart Association.

Between March 2018 and December 2023, 81 patients with ATTRwt-CM were diagnosed. Of these, 20 patients had a NYHA class III or IV and were considered not eligible to initiate tafamidis, while 33 patients started tafamidis but had not completed a 12-month follow-up at the time of the study analysis. Thus, the study population fulfilling the inclusion criteria comprised 28 patients (Table 1). Among these, three patients who died from cardiovascular causes (two due to worsening HF and one from an embolic stroke as the first manifestation of a new onset of atrial fibrillation) were unable to complete the 12-month follow-up and were excluded from the data comparison. The study flow chart is shown in Figure 1.

The final study cohort consisted of 25 patients with ATTRwt-CM, predominantly males (*n* = 23, i.e., 92%), with a mean age of 75.9 ± 6.1 years and a high prevalence of carpal tunnel syndrome (*n* = 21, i.e., 75%). According to the Italian National Regulation for tafamidis prescription, all the patients were in NYHA class I (*n* = 5, i.e., 20%) or II (*n* = 20, i.e., 80%). The cardiac biomarkers, including NT-proBNP and HS-TnI, at baseline were 2045 ± 1196 pg/mL and 89 ± 79 pg/mL, respectively. Most patients were in an early disease stage (stage 1: *n* = 17 (69%) and stage 2: *n* = 7 (28%)), as indicated by the NAC staging system score. The median time between the first evaluation and the evaluation at the time of tafamidis initiation was 3 months (IQR 0–6).

Demographic data and functional and biochemical parameters in patients with ATTRwt-CM at baseline and 12 months after follow-up are summarized in Table 2. The patients treated with tafamidis showed a significant improvement in quality of life according to the KCCQ score (64 ± 20 vs. 75 ± 20, *p* = 0.002) after a 12-month follow-up, while no other statistically significant differences were observed between other clinical, functional, and laboratory parameters.

### 3.2. Echocardiographic Parameters

The baseline and 12-month comprehensive echocardiographic parameters are presented in Table 3. No significant differences were found at the 12-month follow-up timepoint after tafamidis 61 mg treatment, except for a reduction in pulmonary artery systolic pressure (PASP) (34 ± 10 mmHg vs. 30 ± 5 mmHg, *p*-value = 0.008) and an increase in the TAPSE/PASP ratio (0.56 ± 0.24 mm/mmHg vs. 0.63 ± 0.27 mm/mmHg, *p*-value 0.005).

### 3.3. CMR Parameters

CMR imaging examinations were available at baseline and 12 months after treatment in 17 out of 25 patients (68% of the total population). In the remaining eight patients, the exams were not available due to claustrophobia (*n* = 1; 12.5%) or were discarded due to poor image quality caused by artifacts from the device, which limited the consistency of native and post-contrast T1-mapping measurements (*n* = 7, 87.5%). At 12 months of follow-up, a reduction in native T1 time and an improvement in RV volume were observed (Table 4).

### 3.4. Assessment of Disease Progression during 12 Months of Follow-up

Disease progression was assessed according to the recommended measurement tools for detecting ATTRwt-CM progression in patients treated with tafamidis, and the results are shown in Table 5 and Table 6.

After 12 months of treatment with tafamidis 61 mg, clinical progression was observed in six patients, with a prevalence of 24%. In particular, one patient exhibited worsening HF, requiring a significant increase in diuretic treatment, associated with a class increase in the NYHA functional class; one patient experienced worsening HF, necessitating hospitalization, coupled with a significant reduction in 6-MWT. In addition, four patients demonstrated a decrease of at least 30 m in 6-MWT.

Biochemical progression was observed in 13 patients, with a prevalence of 52%. In particular, one patient displayed a significant increase in HS-TnI serum levels; one patient exhibited increases in both HS-TnI and NT-proBNP serum levels; four patients showed at least one stage increase in the NAC staging system score; two patients demonstrated a significant increase in NT-proBNP serum levels; two patients displayed an increase in both the NT-proBNP level and NAC score; and one patient presented all three parameters of biochemical progression.

Structural progression was observed in seven patients, with a prevalence of 28%. In particular, one patient showed an increase in LV wall thickness; one patient experienced a decrease in LVEF; three patients demonstrated an isolated increase in diastolic functioning grade; one patient exhibited an increase in diastolic functioning grade associated with a new onset of atrial fibrillation; and one patient showed a reduction in LVEF associated with an advance in diastolic functioning grade and a new onset of atrial fibrillation.

Considering the presence of at least one parameter for each of the three clinical, biochemical, and structural domains, the overall progression was observed in two patients, with a prevalence of 8% (*n* = 2/25) in our cohort study 12 months after the initial treatment with tafamidis. However, if cardiovascular deaths are considered as events due to disease progression, the final prevalence of disease progression during treatment with tafamidis 61 mg in our cohort of patients with ATTRwt-CM was equal to 18% (*n* = 5/28).

## 4. Discussion

In this study, our aim was to assess the impact of tafamidis 61 mg treatment after 12 months of follow-up in patients with ATTRwt-CM across various parameters, including clinical status, physical performance, quality of life, and functional and structural cardiac remodeling, and to assess the response to treatment in terms of clinical, biochemical, and structural progression using recommended clinical tools for the long-term monitoring of treatment with tafamidis.

Tafamidis, recognized for its ability to reduce cardiovascular mortality and hospitalization due to HF, stands as the first disease-modifying drug approved for treating patients with ATTR-CM and a history of HF [5,8]. The treatment is designed to stabilize the TTR protein, thus preventing its dissociation into monomers, a critical step in fibrillogenesis, and to inhibit the extracellular deposition of amyloid substance, thereby halting the progression of organ damage established by the amyloid burden present at the initiation of treatment.

Monitoring disease progression in patients on tafamidis is challenging and currently lacks best-practice guidance. In our study, we applied a set of clinically feasible tools for the long-term treatment monitoring of patients with ATTR-CM, as suggested by Garcia-Pavia et al. [13]. At the 12-month follow-up timepoint since the start of tafamidis treatment in ATTRwt-CM, only 8% of patients experienced disease progression, suggesting a stabilizing effect of tafamidis treatment on the progressive functional and structural cardiac deterioration typically observed in these patients. Moreover, it was observed that tafamidis treatment in ATTRwt-CM stabilizes functional and structural cardiac parameters, although there is no evidence of favorable reverse remodeling, and the results of the present study are consistent with the post-hoc analysis of the ATTR-ACT trial and recent observational studies.

Specifically, a post-hoc analysis of the ATTR-ACT trial [9] investigated the effect of tafamidis 80 mg treatment on cardiac function over 30 months. The analysis, which focused on changes in echocardiographic parameters such as LVEF, LV SV, LV GLS, and E/E′, demonstrated less pronounced worsening in these measures among patients receiving tafamidis compared to those on placebo, reaffirming the ability of tafamidis treatment to mitigate the decline in LV systolic and diastolic functions over time.

Moreover, Giblin et al. [12] conducted a retrospective study to investigate the impact of tafamidis on myocardial function over 12 months of treatment using serial speckle tracking echocardiography. Their study, comparing 23 patients treated with ATTR-CM to 22 untreated subjects, demonstrated that GLS and MW-derived parameters deteriorated significantly more in the untreated group compared to those treated with tafamidis. Notably, no significant differences were observed between the groups in terms of other echocardiographic parameters, indicating the stabilization effect of tafamidis on myocardial function over one year.

Similarly, Rettl et al. [11] explored the effect of tafamidis treatment on myocardial strain in patients with ATTR-CM. Their study, involving patients treated with tafamidis free acid 61 mg or tafamidis meglumine 20 mg compared to untreated patients, revealed stable measurements in those treated with tafamidis, while untreated patients exhibited significant deterioration in various strain parameters (e.g., LV GLS, RV GLS, and LA reservoir strain). Those differences between the groups (treated vs. untreated) were more pronounced in patients treated with tafamidis free acid 61 mg.

Ichikawa et al. [27] conducted a similar study on 42 patients with ATTR-CM treated with tafamidis and reached the same conclusion. Their subgroup analyses further confirmed the ability of tafamidis treatment to prevent both structural and functional cardiac deterioration over time, irrespective of age and disease stage.

Additionally, Chamling et al. [28] examined the effect of tafamidis treatment after one year of therapy using cardiac MRI. They compared serial multiparametric CMR parameters between treated and untreated patients with ATTRwt-CM. While parameters such as LV wall thickness, LVEF, native T1 time, and ECV values remained unchanged in the tafamidis group, untreated patients exhibited a reduction in LVEF, along with slight increases in LV mass, native T1 time, and ECV. Serum NT-proBNP levels increased in both groups, with a higher increase observed in untreated patients compared to treated ones. Our study revealed a reduction in native T1 time values on cardiac MRI, which was not observed in other studies. Although it may be related to the reduction in inflammatory-based cytotoxic damage and interstitial edema induced by circulating monomers, the cause of this result is unclear.

In the ATTR-ACT trial [8], tafamidis treatment significantly reduced the decline in functional capacity, health status, and quality of life compared to untreated patients. Our study observed different effects of treatment on quality of life compared to those described in the ATTR-ACT trial. While the ATTR-ACT trial showed a reduction in decline, our study demonstrated a significant improvement in QoL after a shorter follow-up period.

These discrepancies could be attributed to differences in the inclusion criteria of the study populations. In the ATTR-ACT trial, patients with a NYHA class ranging from I to III were enrolled, whereas, in our study, patients with NYHA class III were excluded, following the inclusion criteria for tafamidis prescription currently enforced in Italy. The presence of patients at a less advanced stage of the disease could explain the more pronounced positive effect of the treatment on the patients’ quality of life.

### Study Limitations

This study had several limitations. First, this study was not placebo-controlled or randomized. Second, echocardiographic evaluations were performed by a single operator, but intra-observer variability was not specifically assessed. Third, patients were enrolled during a 5-year period, when supportive heart failure and arrhythmias management dramatically changed over time. Fourth, CMR was not performed in nearly one-third of the patients. Finally, it is unclear whether the statistically significant differences in PASP at echocardiography and the T1 value at CMR between baseline and follow-up evaluations are a real phenomenon induced by the treatment with tafamidis or the result of a multiple-comparison problem.

## 5. Conclusions

Patients with ATTRwt-CM typically undergo a progressive decline over time. This study described the impact of tafamidis treatment on clinical, laboratory, and functional parameters. Disease progression, assessed using a multiparametric tool recommended by a recent position paper of experts, was observed in a minority of patients.

## Figures and Tables

**Table 1:** Demographic data and functional, biochemical, and echocardiographic parameters of the study cohort at the baseline evaluation.

Clinical Features	Overall Cohort (*n* = 28)	Patients Experiencing CV Death (*n* = 3)	Final Cohort (*n* = 25)
Demographic data			
Age, years	76.5 ± 6.0	81.0 ± 2.0	75.9 ± 6.1
Male sex	26 (93%)	3 (100%)	23 (92%)
BMI, kg/m^2^	27.8 ± 3.9	26.1 ± 4.6	28.0 ± 3.9
BSA, m^2^	1.81 ± 0.17	1.70 ± 0.10	1.82 ± 0.17
Systolic BP, mmHg	124 ± 14	111 ± 24	126 ± 13
Diastolic BP, mmHg	71 ± 8	66 ± 8	71 ± 8
Atrial fibrillation	10 (36)	3 (100)	7 (28)
Carpal tunnel syndrome	21 (75)	0 (0)	21 (84)
Spinal stenosis	7 (25)	0 (0)	7 (28)
Functional parameters			
NYHA functional class			
I	5 (20)	0 (0)	5 (20)
II	23 (82)	3 (100)	20 (80)
KCCQ	64 ± 20	63 ± 24	64 ± 20
6MWT	310 ± 104	220 ± 105	321 ± 101
NAC stage			
1	19 (68)	2 (67)	17 (68)
2	8 (29)	1 (33)	7 (28)
3	1 (3)	0 (0)	1 (4)
Biochemical parameters			
Creatinine, mg/dL	1.2 ± 0.3	1.4 ± 0.4	1.2 ± 0.3
eGFR, mL/min/1.73 m^2^	59 ± 15	51 ± 22	60 ± 14
K^+^, mEq/L	4.4 ± 0.4	4.8 ± 0.6	4.4 ± 0.3
Na^+^, mEq/L	137 ± 14	135 ± 2	137 ± 15
HS-cTnI, pg/mL	89 ± 75	83 ± 35	89 ± 79
NT-proBNP, pg/mL	2172 ± 1566	3765 ± 4892	2045 ± 1196
Albumin, g/dL	4.2 ± 0.4	3.6 ± 0.9	4.3 ± 0.3
Echocardiographic parameters			
Left ventricle			
LVEDD, mm	48 ± 5	49 ± 3	48 ± 6
LVESD, mm	35 ± 6	41 ± 2	34 ± 6
IVSD, mm	16 ± 2	18 ± 5	16 ± 2
PWD, mm	14 ± 2	15 ± 3	14 ± 2
RWT	0.59 ± 0.12	0.61 ± 0.16	0.59 ± 0.11
LVMi, g/m^2^	174 ± 41	218 ± 65	169 ± 36
LVEDV, mL	101 ± 28	95 ± 37	102 ± 29
LVESV, mL	59 ± 21	61 ± 30	58 ± 21
LVEF, %	43 ± 9	37 ± 8	44 ± 9
MCF, %	15.0 ± 4.8	10.4 ± 4.9	15.5 ± 4.5
3D-LVEDV, mL	108 ± 29	107 ± 26	109 ± 29
3D-LVESV, mL	61 ± 21	70 ± 24	61 ± 21
3D-LVEF, %	44 ± 9	36 ± 8	45 ± 8
3D-LVSV, mL	47 ± 13	37 ± 4	48 ± 13
3D-LMV indexed, g/m^2^	112 ± 24	126 ± 26	110 ± 24
Ea, mmHg	2.4 ± 0.7	2.7 ± 0.3	2.4 ± 0.7
Ees, mmHg	1.6 ± 0.5	1.7 ± 0.7	1.5 ± 0.4
LV GLS, %	−10.5 ± 3.1	−7.5 ± 3.2	−10.8 ± 2.9
EFSR	4.3 ± 0.8	5.4 ± 1.4	4.1 ± 0.6
RRSR	0.9 ± 0.3	1.0 ± 0.2	0.9 ± 0.3
GWI, mmHg%	1080 ± 434	648 ± 457	1128 ± 415
GCW, mmHg%	1256 ± 461	821 ± 573	1307 ± 431
GWW, mmHg%	95 ± 43	98 ± 38	95 ± 44
GWE, %	89 ± 5	85 ± 6	90 ± 4
E wave, cm/s	78 ± 27	116 ± 29	73 ± 24
A wave, cm/s	46 ± 20	55 ± 47	46 ± 18
E/A ratio	1.87 ± 1.07	2.65 ± 1.77	1.78 ± 1.01
DecT, ms	188 ± 47	209 ± 55	186 ± 48
E/E average ratio	14 ± 6	20 ± 9	13 ± 5
Left atrium			
LAD, mm	48 ± 5	45 ± 3	48 ± 5
LAVI, mL/m^2^	49 ± 13	46 ± 11	49 ± 13
LAV pre-P, mL	71 ± 21	61 ± 18	72 ± 22
LAV min, mL	65 ± 21	55 ± 10	66 ± 22
LAPEF, %	16 ± 8	25 ± 2	15 ± 8
LAAEF, %	18 ± 11	7 ± 4	20 ± 10
LAEI, %	39 ± 21	41 ± 4	39 ± 22
TLAEF, %	27 ± 11	29 ± 2	27 ± 11
LACI ratio	3.4 ± 5.5	2.4 ± 0.3	3.5 ± 6.1
LAr_strain (reservoir), %	8.9 ± 5.6	9.3 ± 0.6	8.8 ± 6.0
LAcd_strain (conduit), %	−6.8 ± 3.9	−9.0 ± 1.0	−6.6 ± 4.1
LAc_strain (booster), %	−2.0 ± 2.7	0.3 ± 0.6	−2.3 ± 2.7
Right atrium			
RAA, cm^2^	21.6 ± 5.7	20.4 ± 5.1	21.8 ± 5.8
RAV max, mL	82 ± 29	65 ± 13	84 ± 29
RAV pre-P, mL	60 ± 15	57 ± 22	61 ± 15
RAV min, mL	59 ± 26	54 ± 20	59 ± 27
RAPEF, %	14 ± 7	12 ± 9	14 ± 7
RAAEF, %	24 ± 13	12 ± 15	25 ± 13
RAEI, %	47 ± 29	26 ± 25	50 ± 29
TRAEF, %	29 ± 13	19 ± 15	31 ± 13
RAr_strain (reservoir), %	13 ± 7	8 ± 3	14 ± 7
RAcd_strain (conduit), %	−9 ± 5	−8 ± 4	−9 ± 5
RAc_strain (booster), %	−5 ± 6	−1 ± 0	−5 ± 6
Right ventricle			
RVD1, cm	47 ± 5	46 ± 8	47 ± 5
RVD2, cm	35 ± 6	34 ± 11	35 ± 6
RDV3, cm	74 ± 9	71 ± 14	74 ± 9
3D-RVEDV, mL	109 ± 31	110 ± 42	109 ± 30
3D-RVESV, mL	65 ± 21	74 ± 30	63 ± 21
3D-RVEF, %	41 ± 7	33 ± 4	42 ± 7
3D-RVSV, mL	44 ± 13	36 ± 13	46 ± 13
FAC, %	36 ± 8	31 ± 5	37 ± 8
TAPSE, mm	17 ± 4	14 ± 1	17 ± 4
Tricuspid S wave, cm/s	11 ± 3	12 ± 6	11 ± 3
RV GLS, %	−12 ± 4	−10 ± 4	−13 ± 4
RV FWLS, %	−16 ± 6	−14 ± 6	−16 ± 6
Pulmonary AcT, ms	110 ± 29	96 ± 15	112 ± 30
SPAP, mmHg	35 ± 11	43 ± 14	34 ± 10
TAPSE/SPAP ratio	0.53 ± 0.24	0.33 ± 0.11	0.56 ± 0.24

Abbreviations: 6MWT, 6 min walk test; AcT, acceleration time; BMI, body mass index; BP, blood pressure; BSA, body surface area; DecT, deceleration time; Ea, arterial elastance; Ees, end-systolic elastance; EFSR, ejection fraction on strain ratio; eGFR, estimated glomerular filtration rate; GCW, global constructive work; GWE, global work efficiency; GWI, global work index; GWW, global wasted work; HS-cTnI, high-sensitivity cardiac troponin I; IVSD, interventricular septum diameter; K^+^, serum potassium; KCCQ, Kansas City Cardiomyopathy Questionnaire; LAAEF, left atrial active emptying fraction; LACI, left atrioventricular coupling index; LAD, left atrium dimension; LAEI, left atrial expansion index; LAPEF, left atrial passive emptying fraction; LAVi, left atrial volume indexed; LV GLS, left ventricular global longitudinal strain; LVAC, left ventricular arterial elastance; LVEDD, left ventricular end-diastolic diameter; LVEDV, left ventricular end-diastolic volume; LVEF, left ventricular ejection fraction; LVESD, left ventricular end-systolic diameter; LVESV, left ventricular end-systolic volume; LVMi, left ventricular mass indexed; LVSV, left ventricular stroke volume; MCF, myocardial contraction fraction; Na+, serum sodium; NAC, National Amyloid Center; NT-proBNP, N-terminal pro–B-type natriuretic peptide; NYHA, New York Heart Association; PWD, posterior wall diameter; RAA, right atrial area; RAAEF, right atrial active emptying fraction; RAEI, right atrial expansion index; RAPEF, right atrial passive fraction; RAV, right atrial volume; RRSR, relative regional strain ratio; RVD, right ventricular diameter; RVEDV, right ventricular end-diastolic volume; RVEF, right ventricular ejection fraction; RVESV, right ventricular end-systolic volume; RVSV, right ventricular stroke volume; RWT, relative wall thickness; SPAP, systolic pulmonary artery pressure; TAPSE, tricuspid annular plane systolic excursion; TLAEF, total left atrial emptying fraction; and TRAEF, total right atrial emptying fraction.

**Table 2:** Demographic data and functional and biochemical parameters of the study cohort at baseline and the 12-month follow-up timepoint.

Clinical Features	Baseline (*n* = 25)	Follow-Up (*n* = 25)	Mean Difference	*p*-Value
Demographic data				
Age, years	75.9 ± 6.1	77.1 ± 6.1	1.2 ± 0.1	<0.001
Male sex	23 (92%)	-	-	-
BMI, kg/m^2^	28.0 ± 3.9	27.4 ± 3.6	−0.5 ± 0.4	0.254
BSA, m^2^	1.82 ± 0.17	1.81 ± 0.18	−0.02 ± 0.01	0.256
Systolic BP, mmHg	126 ± 13	128 ± 12	3 ± 2	0.191
Diastolic BP, mmHg	71 ± 8	72 ± 5	1 ± 2	0.613
Atrial fibrillation	7 (28)	10 (40)	-	0.370
Carpal tunnel syndrome	21 (84)	21 (84)	-	1.000
Spinal stenosis	7 (28)	7 (28)	-	1.000
Functional parameters				
NYHA functional class				
I	5 (20)	7 (28)	-	0.574
II	20 (80)	18 (72)	-	
KCCQ	64 ± 20	75 ± 20	11 ± 3	0.002
6MWT	321 ±101	343 ± 116	23 ± 16	0.180
NAC stage				
1	17 (68)	14 (56)	-	0.507
2	7 (28)	8 (32)	-	
3	1 (4)	3 (12)	-	
Biochemical parameters				
Creatinine, mg/dL	1.2 ± 0.3	1.4 ± 0.4	0.2 ± 0.1	0.054
eGFR, mL/min/1.73 m^2^	60 ± 14	55 ± 17	−4 ± 3	0.115
K^+^, mEq/L	4.4 ± 0.3	4.4 ± 0.4	0.0 ± 0.1	0.947
Na^+^, mEq/L	137 ± 15	140 ± 3	4 ± 3	0.247
HS-cTnI, pg/mL	89 ± 79	83 ± 70	−6 ± 21	0.780
NT-proBNP, pg/mL	2045 ± 1196	2575 ± 1711	530 ± 276	0.067
Albumin, g/dL	4.3 ± 0.3	4.3 ± 0.3	−0.0 ± 0.1	0.500
Medical therapy				
Beta-Blockers	5 (20)	10 (40)	-	0.123
ACEi/ARBs	7 (28)	5 (20)	-	0.508
MRA	13 (52)	15 (60)	-	0.568
Furosemide	20 (80)	23 (92)	-	0.221
Furosemide dosage, mg	25 (IQR 13–50)	25 (IQR 25–50)	-	0.187

*Abbreviations*: 6MWT, 6 min walk test; ACEi, angiotensin-converting enzyme inhibitor; ARB, angiotensin receptor blocker; BMI, body mass index; BP, blood pressure; BSA, body surface area; eGFR, estimated glomerular filtration rate; HS-cTnI, high-sensitivity cardiac troponin I; K^+^, serum potassium; KCCQ, Kansas City Cardiomyopathy Questionnaire; MRA, mineralocorticoid receptor antagonist; Na^+^, serum sodium; NAC, National Amyloid Center; NT-proBNP, N-terminal pro–B-type natriuretic peptide; and NYHA, New York Heart Association.

**Table 3:** Morphological and functional echocardiographic parameters of the study cohort at baseline and the 12-month follow-up timepoint.

	Baseline (*n* = 25)	Follow-Up (*n* = 25)	Mean Difference	*p*-Value
Left ventricle				
LVEDD, mm	48 ± 6	48 ± 5	0 ± 0	0.337
LVESD, mm	34 ± 6	33 ± 6	−2 ± 1	0.056
IVSD, mm	16 ± 2	16 ± 2	0 ± 0	1.000
PWD, mm	14 ± 2	14 ± 2	0 ± 0	0.063
RWT	0.59 ± 0.11	0.58 ± 0.11	−0.01 ± 0.01	0.388
LVMi, g/m^2^	169 ± 36	167 ± 32	−2 ± 2	0.524
LVEDV, mL	102 ± 29	103 ± 33	1 ± 4	0.795
LVESV, mL	58 ± 21	57 ± 24	−1 ± 3	0.774
LVEF, %	44 ± 9	45 ± 10	1 ± 1	0.295
MCF, %	15.5 ± 4.5	16.5 ± 5.8	1.0 ± 0.5	0.051
3D-LVEDV, mL	109 ± 29	108 ± 34	1 ± 5	0.870
3D-LVESV, mL	61 ± 21	58 ± 22	−3 ± 7	0.708
3D-LVEF, %	45 ± 8	46 ± 10	1 ± 1	0.357
3D-LVSV, mL	48 ± 13	50 ± 18	2 ± 3	0.486
3D-LMV indexed, g/m^2^	110 ± 24	109 ± 26	−1 ± 2	0.427
Ea, mmHg	2.4 ± 0.7	2.5 ± 0.7	0.2 ± 0.1	0.099
Ees, mmHg	1.5 ± 0.4	1.7 ± 0.4	+0.1 ± 0.1	0.077
LV GLS, %	−10.8 ± 2.9	−10.9 ± 3.0	−0.1 ± 0.3	0.873
EFSR	4.1 ± 0.6	4.3 ± 0.9	0.2 ± 0.1	0.262
RRSR	0.9 ± 0.3	1.0 ± 0.7	0.1 ± 0.1	0.139
GWI, mmHg%	1128 ± 415	1087 ± 340	−40 ± 46	0.391
GCW, mmHg%	1307 ± 431	1289 ± 348	−18 ± 51	0.716
GWW, mmHg%	95 ± 44	96 ± 43	1 ± 7	0.886
GWE, %	90 ± 4	89 ± 4	0 ± 1	0.814
E wave, cm/s	73 ± 24	73 ± 17	0 ± 4	0.933
A wave, cm/s	46 ± 18	48 ± 21	0 ± 3	0.983
E/A ratio	1.78 ± 1.01	1.76 ± 0.92	0.16 ± 0.24	0.502
DecT, ms	186 ± 48	178 ± 53	−8 ± 11	0.505
E/E average ratio	13 ± 5	12 ± 4	−1 ± 1	0.833
Left atrium				
LAD, mm	48 ± 5	48 ± 5	0 ± 0	0.685
LAVI, mL/m^2^	49 ± 13	49 ± 13	0 ± 2	0.796
LAV pre-P, mL	72 ± 22	69 ± 28	−2 ± 4	0.708
LAV min, mL	66 ± 22	65 ± 26	−1 ± 4	0.772
LAPEF, %	15 ± 8	19 ± 7	3 ± 2	0.114
LAAEF, %	20 ± 10	15 ± 10	−6 ± 3	0.055
LAEI, %	39 ± 22	45 ± 28	6 ± 6	0.336
TLAEF, %	27 ± 11	29 ± 12	2 ± 3	0.387
LACI ratio	3.5 ± 6.1	3.2 ± 4.2	−0.3 ± 0.5	0.628
LAr_strain (reservoir), %	8.8 ± 6.0	9.8 ± 5.9	0.9 ± 0.5	0.054
LAcd_strain (conduit), %	−6.6 ± 4.1	−7.4 ± 3.9	−0.9 ± 0.4	0.053
LAc_strain (booster), %	−2.3 ± 2.7	−2.4 ± 2.9	−0.1 ± 0.5	0.872
Right atrium				
RAA, cm^2^	21.8 ± 5.8	21.8 ± 4.6	0.1 ± 0.7	0.890
RAV max, mL	84 ± 29	81 ± 25	−3 ± 3	0.333
RAV pre-P, mL	61 ± 15	59 ± 15	1 ± 1	0.547
RAV min, mL	59 ± 27	55 ± 25	−4 ± 3	0.125
RAPEF, %	14 ± 7	15 ± 9	0 ± 1	0.935
RAAEF, %	25 ± 13	32 ± 11	6 ± 3	0.062
RAEI, %	50 ± 29	58 ± 35	9 ± 5	0.066
TRAEF, %	31 ± 13	34 ± 14	3 ± 2	0.085
RAr_strain (reservoir), %	14 ± 7	11 ± 10	−2 ± 2	0.304
RAcd_strain (conduit), %	−9 ± 5	−8 ± 4	1 ± 1	0.579
RAc_strain (booster), %	−5 ± 6	−5 ± 6	0 ± 1	0.962
Right ventricle				
RVD1, cm	47 ± 5	46 ± 5	1 ± 1	0.265
RVD2, cm	35 ± 6	34 ± 5	0 ± 1	0.575
RDV3, cm	74 ± 9	72 ± 8	−2 ± 1	0.150
3D-RVEDV, mL	109 ± 30	113 ± 29	4 ± 4	0.381
3D-RVESV, mL	63 ± 21	66 ± 20	3 ± 3	0.381
3D-RVEF, %	42 ± 7	41 ± 9	−1 ± 1	0.579
3D-RVSV, mL	46 ± 13	47 ± 16	1 ± 2	0.603
FAC, %	37 ± 8	37 ± 9	1 ± 1	0.561
TAPSE, mm	17 ± 4	18 ± 5	1 ± 1	0.216
Tricuspid S wave, cm/s	11 ± 3	12 ± 2	0 ± 0	0.737
RV GLS, %	−13 ± 4	−13 ± 4	1 ± 0	0.335
RV FWLS, %	−16 ± 6	−17 ± 5	1 ± 1	0.184
Pulmonary AcT, ms	112 ± 30	115 ± 28	3 ± 2	0.210
SPAP, mmHg	34 ± 10	30 ± 5	−5 ± 2	0.008
TAPSE/SPAP ratio	0.56 ± 0.24	0.63 ± 0.27	−0.07 ± 0.02	0.005

Abbreviations: AcT, acceleration time; DecT, deceleration time; Ea, arterial elastance; Ees, end-systolic elastance; EFSR, ejection fraction on strain ratio; GCW, global constructive work; GWE, global work efficiency; GWI, global work index; GWW, global wasted work; IVSD, interventricular septum diameter; LAAEF, left atrial active emptying fraction; LACI, left atrioventricular coupling index; LAD, left atrium dimension; LAEI, left atrial expansion index; LAPEF, left atrial passive emptying fraction; LAVi, left atrial volume indexed; LV GLS, left ventricular global longitudinal strain; LVAC, left ventricular arterial elastance; LVEDD, left ventricular end-diastolic diameter; LVEDV, left ventricular end-diastolic volume; LVEF, left ventricular ejection fraction; LVESD, left ventricular end-systolic diameter; LVESV, left ventricular end-systolic volume; LVMi, left ventricular mass indexed; LVSV, left ventricular stroke volume; MCF, myocardial contraction fraction; PWD, posterior wall diameter; RAA, right atrial area; RAAEF, right atrial active emptying fraction; RAEI, right atrial expansion index; RAPEF, right atrial passive fraction; RAV, right atrial volume; RRSR, relative regional strain ratio; RVD, right ventricular diameter; RVEDV, right ventricular end-diastolic volume; RVEF, right ventricular ejection fraction; RVESV, right ventricular end-systolic volume; RVSV, right ventricular stroke volume; RWT, relative wall thickness; SPAP, systolic pulmonary artery pressure; TAPSE, tricuspid annular plane systolic excursion; TLAEF, total left atrial emptying fraction; and TRAEF, total right atrial emptying fraction.

**Table 4:** Morphological and functional cardiac magnetic resonance parameters of the study cohort at baseline and the 12-month follow-up timepoint.

	Baseline (*n* = 17)	Follow-Up (*n* = 17)	Mean Difference	*p*-Value
LVEDV indexed, mL/m^2^	75 ± 25	71 ± 19	−3 ± 2	0.258
LVESV indexed, mL/m^2^	33 ± 20	28 ± 13	−5 ± 2	0.056
LVSV index, mL/m^2^	42 ± 11	44 ± 11	2 ± 2	0.236
LVEF, %	57 ± 12	62 ± 11	4 ± 2	0.040
LV MWT, mm	19 ± 3	19 ± 3	0 ± 0	0.886
T1-time, ms	1162 ± 66	1116 ± 52	−43 ± 11	0.001
ECV, %	51 ± 8	50 ± 10	0 ± 0	0.886
LAA, cm^2^	31 ± 6	30 ± 6	−1 ± 1	0.170
RVEDV indexed, mL/m^2^	68 ± 14	60 ± 13	−8 ± 2	0.004
RVESV indexed, mL/m^2^	26 ± 10	22 ± 7	−4 ± 1	0.017
RVSV indexed, mL/m^2^	42 ± 10	38 ± 12	−4 ± 2	0.039
RVEF, %	62 ± 10	63 ± 11	1 ± 2	0.456
RAA, cm^2^	24 ± 7	23 ± 8	0 ± 1	0.637

Abbreviations: ECV, extracellular volume; LAA, left atrial area; LV MWT, left ventricular maximal wall thickness; LVEDV, left ventricular end-diastolic volume; LVEF, left ventricular ejection fraction; LVESV, left ventricular end-systolic volume; LVSV, left ventricular stroke volume; RAA, right atrial area; RVEDV, right ventricular end-diastolic volume; RVEF, right ventricular ejection fraction; RVESV, right ventricular end-systolic volume; and RVSV, right ventricular stroke volume.

**Table 5:** Proportion of patients with disease progression during 12 months of follow-up.

Tool and Domain	Clinical Feature	Threshold Indicating Disease Progression	Disease Progression
Clinical progression (clinical and functional parameters)			6 (24)
Clinical and medical history	Cardiovascular-related hospitalization	Heart failure-related hospitalization	2 (8)
NYHA class	Stepwise class change	One class increase in NYHA	1 (4)
KCCQ	Description of measurements	10-point decrease in KCCQ	0 (0)
Functional capacity	6MWT	Decrease of 30–40 meters (in the absence of obvious non-cardiovascular causes)	5 (20)
Biochemical progression (biomarkers and laboratory markers)			13 (52)
Biomarkers and laboratory markers	NT-proBNP	30% increase with 300 pg/mL cut-off	7 (28)
	Troponin (high-sensitivity) assay	30% increase	3 (12)
	Clinical staging system	Advance in NAC staging score	9 (36)
Structural progression (imaging parameters and ECG)			7 (28)
Echocardiography	LV measures wall thickness/mass	≥2 mm increase in LV MWT	1 (4)
	Systolic function measurements	≥5% decrease in LVEF	2 (8)
		≥5 mL decrease in SV and ≥1% increase in LV GLS	0 (0)
	Diastolic dysfunction worsening	Stepwise increase in diastolic functioning grade	5 (20)
Electrocardiography/ Holter ECG	New onset of arrhythmic/conduction disturbances	New onset of bundle branch block	0 (0)
		New onset of atrioventricular block	0 (0)
		New onset of arrhythmias with an indication of permanent pacing (sinus node dysfunction or atrial fibrillation with a very slow ventricular response without pharmacological treatment)	3 (12)
Overall disease progression (at least one criterion from each domain: clinical; biochemical; and structural)			2 (8)

Abbreviations: 6MWT, 6 min walk test; ECG, electrocardiography; KCCQ, Kansas City Cardiomyopathy Questionnaire; LV, left ventricular; MWT, maximal wall thickness; NAC, National Amyloid Center; NT-proBNP, N-terminal pro–B-type natriuretic peptide; and NYHA, New York Heart Association.

**Table 6:** Patients with clinical, biochemical, and structural progression during 12 months of follow-up.

	Clinical and Functional Endpoints				Biomarkers and Laboratory Markers			Imaging and Electrocardiographic Parameters					
	CV-RH	NYHA Class Increase	KCCQ Score Decrease	6MWT Decrease	NT-proBNP Increase	HS-cTn Increase	Advance in NAC Score	LVWT Increase	LVEF Decrease	LV SV Decrease/GLS Increase	Advance in Diastolic Functioning Grade	New Onset of Arrhythmic or Conduction Disorders	Progression
ID1	0	0	0	0	0	0	1	0	0	0	0	0	0
ID2	0	0	0	1	0	0	0	0	1	0	0	0	0
ID3	0	0	0	0	1	0	1	0	0	0	0	0	0
ID4	0	0	0	0	1	0	0	0	0	0	0	0	0
ID5	0	0	0	0	1	0	1	0	0	0	0	0	0
ID6	0	0	0	0	0	0	0	0	0	0	0	0	0
ID7	1	0	0	1	0	0	1	1	0	0	0	0	1
ID8	0	0	0	0	0	0	0	0	0	0	1	1	0
ID9	0	0	0	0	0	0	1	0	0	0	1	0	0
ID10	0	0	0	0	0	0	1	0	0	0	0	0	0
ID11	0	0	0	0	0	0	1	0	0	0	1	0	0
ID12	0	0	0	1	0	0	0	0	0	0	0	0	0
ID13	0	0	0	0	0	0	0	0	0	0	0	0	0
ID14	0	0	0	0	0	0	0	0	0	0	0	1	0
ID15	0	0	0	0	0	0	0	0	0	0	0	0	0
ID16	0	0	0	0	0	0	0	0	0	0	0	0	0
ID17	0	0	0	0	0	0	0	0	0	0	0	0	0
ID18	0	0	0	1	0	0	0	0	0	0	0	0	0
ID19	0	0	0	1	1	0	0	0	0	0	0	0	0
ID20	1	1	0	0	1	1	1	0	0	0	1	0	1
ID21	0	0	0	0	0	0	0	0	1	0	1	1	0
ID22	0	0	0	0	0	1	0	0	0	0	0	0	0
ID23	0	0	0	0	1	0	1	0	0	0	0	0	0
ID24	Deceased												
ID25	Deceased												
ID26	0	0	0	0	1	1	0	0	0	0	0	0	0
ID27	0	0	0	0	0	0	0	0	0	0	0	0	0
ID28	Deceased												

*Abbreviations*: 6MWT, 6 min walk test; CV-RH, cardiovascular-related hospitalization; GLS, global longitudinal strain; HS-cTnI, high-sensitivity cardiac troponin I; LV, left ventricular; LVWT, left ventricular wall thickness; NAC, National Amyloid Center; N-terminal pro–B-type natriuretic peptide; NYHA, New York Heart Association; and SV, stroke volume.

## Data Availability

Data supporting the results of this study are available from the corresponding author upon reasonable request.
